# Therapeutic effects of olive leaf tea (*Olea europaea L.*) on gastrointestinal symptoms and body composition in adults with small intestinal bacterial overgrowth

**DOI:** 10.3389/fnut.2025.1659500

**Published:** 2025-08-22

**Authors:** Ayesha Zafar, Waqas Ahmed, Sajid Khan Tahir, Abdul Momin Rizwan Ahmad, Sanaullah Iqbal

**Affiliations:** ^1^Department of Food Science and Human Nutrition, University of Veterinary and Animal Sciences, Lahore, Pakistan; ^2^Department of Physiology, University of Veterinary and Animal Sciences, Lahore, Pakistan; ^3^Department of Health Sciences, University of York, York, United Kingdom; ^4^Department of Human Nutrition and Dietetics, NUST School of Health Sciences, National University of Sciences and Technology (NUST), Sector H-12, Islamabad, Pakistan

**Keywords:** gastrointestinal symptoms, hydrogen breath test, olive leaf tea, SIBO, gut microbiota

## Abstract

**Introduction:**

Olive leaf has strong antibacterial and anti-inflammatory properties, potentially modulating gut microbiota composition. This may help address small intestinal bacterial overgrowth (SIBO), a gastrointestinal (GI) problem causing malabsorption and potential complications.

**Objective:**

This study aimed to observe the effect of olive leaf tea (OLT) on GI symptoms, body composition, and the hydrogen/methane breath test among patients suffering from SIBO.

**Methods:**

A total of 49 SIBO individuals, confirmed after a glucose breath test (GBT), were divided randomly into two groups. The treatment group (*n* = 25) took OLT (1.7 g leaf powder/250 mL water) twice/day for 2 months, and the control group (*n* = 24) was given no tea. After the intervention, GBT was conducted, and symptoms were assessed through the GI symptom rating scale (GSRS) and symptomatic questionnaire, and body composition parameters were assessed. The area under the curve, chi-square, independent, and paired sample *t*-tests were performed for data analysis.

**Results:**

In the intervention group, there was a significant decrease observed in GSRS score (from 19 to 6.8), symptomatic score (4.1 to 1.7), H_2_/CH_4_ peak (20.8–5.7 ppm), mean H_2_ (*p* = 0.0041) and mean H_2_ + CH_4_ production (*p* = 0.0043), with 88% GBT normalization rate (*p* = 0.001), as compared to the control group. A significant decrease in weight, TBW, BMR, and muscle mass was also documented (*p* < 0.05).

**Conclusion:**

This study concludes that OLT consumption might have therapeutic benefits against SIBO by alleviating symptoms and normalizing GBT, but does not significantly improve body composition parameters.

## 1 Introduction

Small intestinal bacterial overgrowth (SIBO) is a GI problem that can be described as colonization of opportunistic or pathogenic bacteria in the small intestine (1). SIBO remained undiscovered or misdiagnosed for many years because its clinical symptoms overlap with other diseases such as celiac disease, *Helicobacter pylori*, and inflammatory bowel disease (IBS). The treatment of SIBO is challenging, especially in non-symptomatic patients. Therefore, SIBO remains untreated until it causes much damage to the small intestine and presents with further complications (2). Manifestations of SIBO mainly include abdominal pain, distension, bloating, cramping, flatulence, diarrhea, and constipation (2). Risk factors for SIBO include older age, excessive use of PPIs and antibiotics, hypo-chlorhydria, motility disorder, and IBS (3).

Researchers were unable to provide a definitive estimate of SIBO prevalence in the general population. A few studies reported 2.5–22% prevalence in healthy individuals (4), 4.42% in healthy adults in Lahore, Pakistan (5), 31.5% in suspected patients with GI distress, 36.4% in patients with IBS (6), 20% in asymptomatic patients, 50–60% in patients with pancreatitis, 60% in patients with gastroparesis, 9–55% in patients with celiac disease, and 59% in patients with diverticulitis (5).

SIBO has two subtypes, which are classified based on the ratio of gasses produced by the microorganisms residing in the small intestine. Hydrogen-dominant SIBO patients often present with diarrhea, and methane-dominant ones experience decreased bowel transit time, slow basal metabolic rate, and delayed gastric emptying (7). The gut of a healthy person harbors Bacteroidetes and Firmicutes in a balanced ratio, accounting for 90% of the gut microbiome. A comparative analysis of SIBO patients revealed a higher abundance of Firmicutes. Common bacteria that contaminate the gut in SIBO include *Escherichia coli, Enterococcus* spp.*, Klebsiella pneumoniae,* and *Pseudomonas aeruginosa* (8).

There are three recommended approaches to manage or cure SIBO. First, addressing the underlying cause; second, eradicating bacterial overgrowth; and third, overcoming nutritional deficiencies. Antibiotics are considered the mainstay therapy for SIBO. Taking antibiotics alone increases the chances of recurrence of symptoms, side effects, and causes antibiotic resistance. Herbal therapy is considered as effective as Rifaximin (an antibiotic used to treat IBS with diarrhea) in treating SIBO. Approximately 57% of SIBO patients who did not respond to antibiotics showed a positive response to herbal therapy (9). Therefore, this is the need of the hour to uncover functional foods that may have the potential to manage or treat SIBO.

Olive trees (*Olea europaea L.*), belonging to the Oleaceae family, grow in places with a Mediterranean atmosphere and are well-adapted to arid conditions. They are dark green and oblong with tapered ends (10). They tend to have a mild bitter taste, attributed to their compound oleuropein. The historical use of olive oil and leaves as natural healers is extensively documented in religious texts. Olive leaves infusion contains antioxidants, including oleuropein, quercetin, fatty acids (oleic acid and oleanolic acid), and the most potent antioxidant hydroxytyrosol and its derivatives. The phenolic content of olive leaves is higher than extra virgin olive oil and olive fruit (11). The olive leaves’ biophenols exhibit strong antimicrobial, antioxidant, and anti-inflammatory properties, also flourish the healthy gut microbiota, and decrease the likelihood of bowel-related disorders (12). Ascribed to this, olive leaves may prove beneficial in managing health problems related to bacterial contamination and inflammation. The current study was designed to assess the impact of OLT on intestinal health, GI symptoms, the concentration of H_2_ and CH_4_ gasses in exhaled breath, and to analyze the OLT impact on the body composition parameters of SIBO patients.

## 2 Materials and methods

### 2.1 Study design

The study was conducted as a randomized controlled trial. The selection of a 2-month (~ 9 weeks) intervention time was based on a previous 10-week clinical study evaluating the therapeutic potential of an antimicrobial herbal blend against SIBO. A trial investigating the effects of the same OLT on hematological parameters also used a 6–12-week duration, and a significant increase in hematocrit was observed in 6 weeks. The methodology of the study consisted of five phases. **Phase 1:** processing and preparation of Olive Leaf Tea (OLT), **Phase 2:** screening to identify SIBO suspected individuals, **Phase 3:** confirmation of SIBO through the hydrogen breath test using a breath analyzer, **Phase 4:** provision of intervention, and **Phase 5:** final assessment after intervention.

### 2.2 Olive leaf tea (OLT) processing and preparation

**Figure 1:**
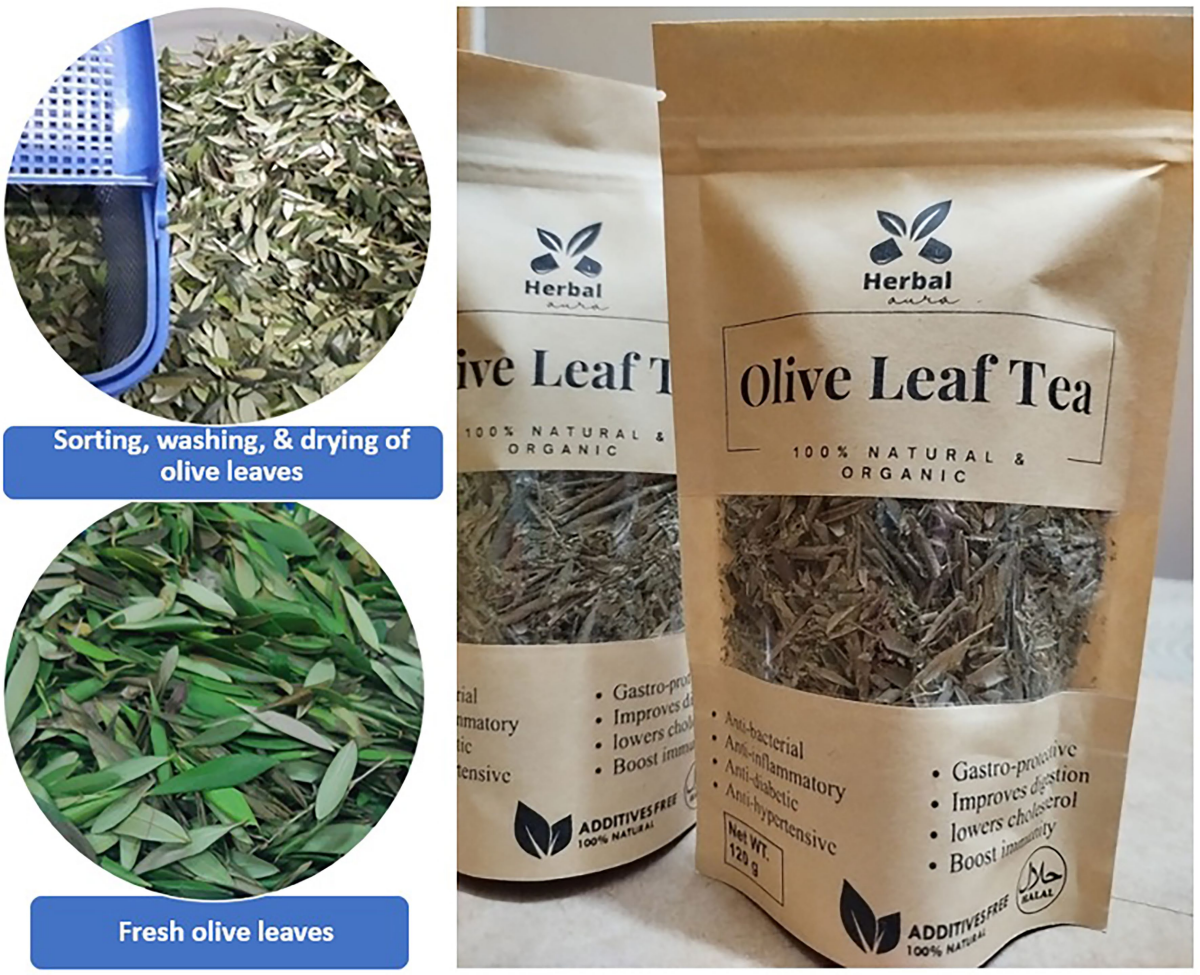
Preparation of packaged olive leaf tea.

Fresh olive leaves were purchased from the district Chakwal, Pakistan, harvested in the morning, during September, the non-blooming season, to ensure maximum phytochemical content. The leaves were sorted, rinsed with clean water, and dried at 45°C in the hot air oven (POL-EKO APARATURA® Model: SLN53) until the moisture content was reduced to 12% or less (Figure 1), measured using a Halogen Moisture Analyzer (13). The leaves were crushed and then incorporated into airtight tea packets. No sugar or sweetener was added during the packaging and brewing process of tea. The OLT was evaluated for sensory evaluation by a semi-trained panel. The judges were asked to rate the provided OLT for its aroma, flavor, and color, and overall acceptability on a 9-point hedonic scale ranging from (1 = dislike extremely) and (9 = like extremely).

### 2.3 Screening and recruitment of participants for intervention

Study participants were recruited via circulated questionnaires across different university campuses. This screening symptomatic questionnaire was developed based on Erdogan et al. (2) with minor modifications. In the questionnaire, individuals were asked to rate the intensity, frequency, and duration of their nine GI symptoms (bloating, burping, diarrhea, nausea, vomiting, feeling of satiety, constipation, abdominal pain, and indigestion) using a 3-point Likert scale. Individuals with a mean score ≥4 were considered suspected of SIBO and were further referred to GBT for confirmation. Participants were also asked to indicate foods that initiate or trigger their GI symptoms.

### 2.4 Confirmatory hydrogen and methane breath test

The participants were instructed to avoid antibiotic use 1 month prior, laxative and PPI use 1 week prior, and fermentable carbohydrate and dairy diet 1–2 days before the test. The 8–10 h of fasting were recommended before the test, while participants can drink water. It was advised to avoid smoking 2 h before the test and to rinse the mouth with antiseptic mouthwash before the test. The breath test was done at the time of screening using a QuinTron breath analyzer (Model SC, QuinTron Instrument Company, Milwaukee, Wisconsin, USA) following instructions given in the manual. The calibration of the analyzer was performed using 20 mL of Quin gas with standard concentrations of H_2_ = 154 ppm, CH_4_ = 74 ppm, and CO_2_ = 6.1%.

On the test day, the baseline reading of the breath sample was taken empty stomach, and then 50 g of glucose dissolved in 250 mL of water was given to participants orally. Breath samples were taken every 15 min for 2 h, and the concentration of gasses in the breath sample was measured. Individuals having at least a 12 ppm rise in H_2_ and/or a ≥ 10 ppm rise in CH_4_ from the baseline reading or the lowest preceding value were diagnosed as positive for SIBO (6).

#### 2.4.1 Inclusion and exclusion criteria

The adults aged 18 years or older and with a mean symptomatic score ≥4 and positive GBT were included in the study. The participants who were hospitalized, diabetic, with chronic medical problems like stroke, coronary obstructive pulmonary disease (COPD), and cancer, with a history of GI surgeries except for cholecystectomy, hysterectomy, and appendectomy, and who were taking antibiotics in the last month were excluded from the study.

#### 2.4.2 Assessment of body composition parameters

Body composition was assessed using the InBody Analyzer 270. Recommendations were to avoid exercise 6–12 h before the test and to stay hydrated and remove socks, metal objects, and heavy clothing (jacket) before the test.

The InBody report provides information about total body water, muscle content, basal metabolic rate, and obesity degree (waist-to-hip ratio, visceral fat, body mass index, and percent body fat), etc. BMI categories were classified according to Asian-Pacific recommendations as “underweight” (< 18.5 kg/m^2^), “normal” (18.5–22.9 kg/m^2^), “overweight” (23–24.9 kg/m^2^), and “obese” (25–29.9 kg/m^2^), and morbid obese (≥30 kg/m^2^).

#### 2.4.3 Assessment of GI symptoms

Patients filled a self-administered validated Gastrointestinal Symptom Rating Scale (GSRS) questionnaire consisting of 15 items categorized into five syndromes: abdominal pain, dyspepsia, indigestion, diarrhea, and constipation. Each category has 3 sub-dimensions, i.e., intensity (none, mild, moderate, and severe), frequency (none, 1–2 times/month, once a week, and >1 time/week), and duration (none, <10 min, 10–30 min, and >30 min). These dimensions were assessed using a 4-point Likert scale (0 = no symptoms; 1 = mild discomfort; 2 = moderate discomfort; 3 = severe discomfort). The total GSRS score was 45, and the mean GSRS was 9.

### 2.5 Provision of intervention

After written agreement, 50 participants with positive BT results were randomly assigned to two groups, with 25 participants in each group: treatment (*n* = 25) and control (*n* = 25). This grouping was performed using the online Research Randomizer software. One participant from the control group left during the study duration.

The intervention group was instructed to take OLT twice a day for 2 months. The participants were instructed to immerse 1.7 g of leaf powder (2 tsp) in hot water for 7–10 min for a better infusion (14). Participants were asked to document their OLT intake daily, as well as any negative effects they may have encountered. The control group did not receive OLT or any other intervention.

### 2.6 Final assessment

At the end of the 2-month study period, the final readings of GBT and body composition analysis of both intervention and control groups were taken. The participants were also requested to fill out the GSRS questionnaire and the 9-symptoms questionnaire to analyze any improvement in the severity of their GI symptoms.

### 2.7 Statistical analysis

SPSS (version 23; SPSS Inc., Chicago, IL, USA) was used for statistical data analysis. The comparison between baseline and termination point readings of mean symptomatic score, GSRS score, GBT normalization rate, and body composition variables was carried out using a paired sample *t*-test. An independent sample *t*-test was applied to compare the above parameters between the control and intervention groups. Mean H_2_ and CH_4_ gas concentrations in the breath sample were calculated using IUAC. The age, gender, and BMI were correlated with SIBO using a Pearson chi-square test. For all analyses, a significance level of *p* < 0.05 was applied.

## 3 Results

This study includes 49 individuals diagnosed with SIBO, with 43 women and 6 men. Table 1 provides a comparison of the demographic characteristics between the control and treatment groups. The age distribution was almost similar across all SIBO groups, with a predominant representation of females in each group. Out of 49 total participants, the majority of the participants belonged to the normal BMI category (38%), followed by the obese (30.6%). The prevalence of morbidly obese patients was low, with 8% reported in the intervention group and 4.2% in the control group.

**Table 1:** Characteristics of SIBO patients.

Participants’ characteristics	*N* (Total)	Control (*n* = 24)	Intervention (*n* = 25)	*p-*value
Age	49	22.4 ± 2.2^a^	24.9 ± 9.1	0.194
Gender				
Female	43 (88%)^b^	22 (91%)	21 (84%)	0.879
Male	6 (12%)	2 (8%)	4 (16%)	0.414
*p*-value	0.000*	0.000*	0.001*	
BMI Groups				
Underweight	4 (8.1%)	3	1	0.317
Normal	19 (38%)	10	9	0.819
Overweight	8 (16.3%)	4	4	1.000
Obese	15 (30.6%)	6	9	0.439
Morbid obese	3 (6.1%)	1	2	0.564
*p*-value	0.000*	0.045*	0.021*	

^a^ Mean ± SD. ^b^ *N* (%), descriptive statistics, chi-square and cross tabs applied,*significant parameter.

The sensory evaluation results, on a 9-point hedonic scale, show that the OLT secured very good results with a mean score of 7 ± 1.2 (like extremely, 11.5%; like very much, 31%, and moderately like 27%) while none of the judges disliked the product.

Table 2 shows the prevalence of SIBO subtypes (H_2_ positive, CH_4_ positive, and H_2_/CH_4_ positive) in the control and treatment groups, and also the effectiveness of OLT intervention in reducing SIBO. The majority, 40.8% (20/49) of patients in both groups at baseline, were H_2_^+^ producers, and 34.6% (17/49) were CH_4_ producers, whereas 24.4% (12/49) of the participants were both H_2_ and CH_4_ producers. After 2 months of intervention, methane-dominant SIBO patients decreased from 36 to 8% in the intervention group, while increasing from 33.4 to 45.8% in the control group. In the treatment group, 88% of SIBO patients showed negative hydrogen and methane breath tests, while only 4.2% showed negative HBT in the control group.

**Table 2:** The percentage of different types of SIBO patients in both groups.

Groups	Time	H_2_^+^	CH_4_^+^	H_2_/CH_4_^+^	Negative	*p*-value
Control *n* = 24	Before	11 (45.8)	8 (33.4)	5 (20.8)	0 (0)	0.233
	After	10 (41.6)	11 (45.8)	2 (8.3)	1 (4.2)	
Treatment *n* = 25	Before	9 (36)	9 (36)	7 (28)	0 (0)	0.001*
	After	1 (4)	2 (8)	0 (0)	22 (88)	

*n* (%), chi-square. *significant parameter.

The comparison of the three subtypes of SIBO based on the mean production of H_2_ and CH_4_ gasses is shown in Table 3. The total body protein, bone minerals, fat-free mass (FFM), skeletal muscle mass (SMM), and basal metabolic rate (BMR) were significantly deficient in CH_4_ producers. The SMM, FFM, total body water (TBW), and BMR in the H_2_^+^/CH_4_^+^-dominant patients were substantially higher than other groups (*p* = 0.036, *p* = 0.038, *p* = 0.041, and *p* = 0.041, respectively).

**Table 3:** Comparison of body composition parameters in different types of SIBO patients.

Parameters	H_2_^+^	CH_4_^+^	H_2_/CH_4_^+^	*p*-value
Weight (kg)	60.97 ± 6.97	60.51 ± 10.17	68.69 ± 17.25	0.341
Total Body Water (kg)	28.44^ab^ ± 3.99	26.14^b^ ± 2.13	33.8^a^ ± 8.96	0.041*
Protein (kg)	7.58^ab^ ± 1.08	6.88^b^ ± 0.43	9.11^a^ ± 2.53	0.030*
Minerals (kg)	2.8^ab^ ± 0.36	2.58^b^ ± 0.2	3.25^a^ ± 0.79	0.043*
Body Fat Mass (BFM)	22.16 ± 6.84	24.81 ± 8.58	22.53 ± 7.89	0.758
Fat Free Mass (FFM)	38.81^ab^ ± 5.43	35.61^b^ ± 2.61	46.16^a^ ± 12.26	0.038*
Skeletal Muscle Mass (SMM)	20.91^ab^ ± 3.32	18.95^b^ ± 1.52	25.47^a^ ± 7.54	0.036*
Body Mass Index (BMI)	23.83 ± 3.55	24.83 ± 4.58	24.47 ± 3.9	0.876
Percentage Body Fat (PBF)	36.01 ± 8.87	40.01 ± 7.5	32.64 ± 6.44	0.209
In-Body Score (IBS)	65.33 ± 4.82	62.88 ± 7.18	67 ± 7.87	0.487
Basal Metabolic Rate (BMR)	1208^ab^ ± 117	1141^b^ ± 60	1366^a^ ± 265	0.041*
Waist to Hip Ratio (WHR)	0.87 ± 0.06	0.86 ± 0.04	0.9 ± 0.06	0.399
Visceral Fat Level (VFL)	10.67 ± 4.36	10.88 ± 5	10 ± 4.32	0.929
Obesity Degree (OD)	110.6 ± 16.7	115.13 ± 20.63	112.57 ± 17.22	0.881
Abdomen Circumference (AC)	82.41 ± 8.93	82.38 ± 9.78	87.63 ± 12.42	0.538
Skeletal Muscle Index (SMI)	6.11 ± 0.65	5.84 ± 0.5	6.89 ± 1.47	0.103
Skeletal Muscle Mass/ Weight	34.46 ± 5.21	32.11 ± 3.82	36.97 ± 4	0.133
Fat Free Mass Index (FFMI)	15.03 ± 1.42	14.68 ± 1.11	16.46 ± 3.05	0.205
Fat Mass Index (FMI)	8.78 ± 3.1	10.1 ± 3.62	7.99 ± 2.14	0.411

Mean ± SD, ANOVA. *significant parameters (level of significance 95%). The parameters having different superscript letters in a row are statistically significant to each other.

The high fat percentage is directly correlated with increased concentrations of exhaled CH_4_, but the difference did not reach the significance level. Similarities were seen in three groups regarding visceral fat level and degree of obesity; however, there was a trend toward higher total body protein (kg) (*p* = 0.030), bone minerals (*p* = 0.043), and weight (*p* = 0.341) observed in the H_2_^+^/CH_4_^+^-group.

Table 4 presents the parameters that show significant changes during the study period. Notably, the treatment group exhibited a decreasing trend in TBW. Although a decrease in BMI (*p* = 0.376) and obesity degree (*p* = 0.475) has been noticed with regular consumption of OLT, these differences did not reach statistical significance. This post-intervention decrease in body weight and BMI, and BMR may be attributed to a significant decrease in TBW, FFM (*p* = 0.024), SMM (*p* = 0.013), and skeletal muscle index (SMI) (*p* = 0.000) in the treatment group. Conversely, the control group showed a statistically significant increase in TBW, FFM, SMM, BMR, FFMI, and SMI.

**Table 4:** Change in body composition parameters between control and treatment groups after Intervention of OLT for 2 months.

Body composition parameters	Control group	Treatment group	*p*-value
Weight (kg)	0.5 ± 2.1	−0.2 ± 2.8	0.369
TBW (kg)	0.1 ± 0.5	−0.4 ± 0.8	0.005*
Protein (kg)	0 ± 0.2	−0.1 ± 0.3	0.386
Minerals (kg)	0 ± 0.1	0 ± 0.1	0.707
Body Fat Mass (BFM)	0.3 ± 2	0.4 ± 2.3	0.992
Fat Free Mass (FFM)	0.1 ± 0.6	−0.5 ± 1.1	0.024*
Skeletal Muscle Mass (SMM)	0.1 ± 0.4	−0.3 ± 0.6	0.013*
Body Mass Index (BMI)	0.2 ± 0.8	−0.1 ± 1.1	0.376
Percentage Body Fat (PBF)	0.4 ± 2.4	0.7 ± 2.5	0.638
In-Body Score (IBS)	−0.1 ± 2.2	−1 ± 2.4	0.169
Basal Metabolic Rate (BMR)	3.1 ± 13.3	−11.2 ± 21.8	0.008*
Waist to Hip Ratio (WHR)	0 ± 0	0 ± 0	0.087
Visceral Fat Level (VFL)	0.2 ± 1.3	0.8 ± 2.2	0.26
Obesity Degree (OD)	0.7 ± 3.8	−0.2 ± 5.3	0.475
Abdomen Circumference (AC)	0.3 ± 2	1.1 ± 3.7	0.303
Skeletal Muscle Index (SMI)	0.1 ± 0.1	−0.1 ± 0.2	0.000*
Skeletal Muscle Mass/ Weight	−0.1 ± 1.4	−0.5 ± 1.4	0.314
Fat Free Mass Index (FFMI)	0.1 ± 0.2	−0.2 ± 0.4	0.011*
Fat Mass Index (FMI)	0.1 ± 0.8	0.2 ± 0.9	0.775

*Mean difference ±S.D., independent sample *t*-test applied (level of significance 95%).

Table 5 presents the comparison of baseline and post-study breath test readings among the three SIBO subtypes based on the median production of H_2_ and CH_4_ gasses, illustrated as the incremental area under the curve (IAUC) (ppm/min), measured during the 90-min breath test after glucose administration. In the control group, the concentration of methane showed a significant rise after 2 months. However, in mixed-type SIBO, a significant increase has been observed in the median production of hydrogen and methane gasses (*p* = 0.931). After 2 months of OLT intervention, a significant decrease was observed in the concentration of H_2_ and CH_4_ gasses in mixed (H_2_/CH_4_) SIBO type (*p* = 0.043). The level of exhaled H_2_ gas in the H_2_-dominant group also showed a considerable decline (*p* = 0.041). Methane gas production in the intervention group also decreased compared to the baseline, but this decline did not reach a significant level (*p* = 0.877).

**Table 5:** Within and between groups comparison of mean hydrogen and methane gas as area under the curve (ppm/min) in H_2_ and CH_4_ type SIBO.

Groups	Interval	H_2_ dominant	CH_4_ dominant	H_2_ + CH_4_ dominant
Control	Before	1,951.4 ± 2,189.9	305.7 ± 297.8	1,014.4 ± 926.4
	After	432.4 ± 414.4	1,038.2 ± 1,648.3	1,087.6 ± 1,336.5
	*p* value	0.092	0.357	0.931
Intervention	Before	1,808.9 ± 2,147.3	211.9 ± 263.2	1,417.2 ± 1,195.1
	After	66.7 ± 94.0	190.9 ± 269.3	257.4 ± 377.2
	*p* value	0.041*	0.877	0.0431*

Data is represented as IUAC ppm/min ± S.D. Values calculated with the use of an independent sample *t*-test, *level of significance (95%).

**Figure 2:**
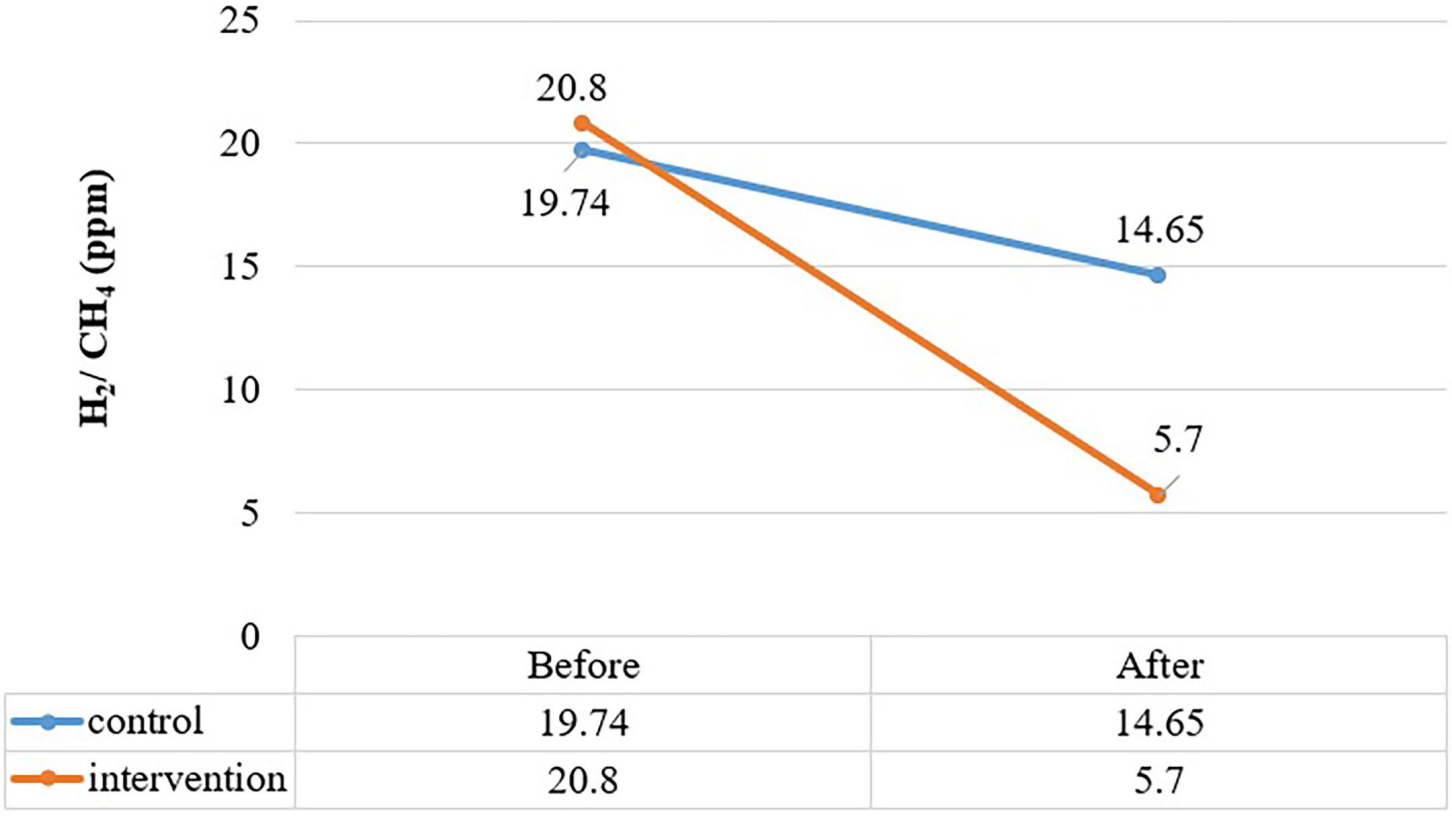
Comparition of peak rise in H2 and CH2 (ppm) between control and treatment group at baseline and end intervention.

Figure 2 shows the peak hydrogen and methane production in subjects calculated during 90-min breath testing. In the control group, the peak mean of H_2_/CH_4_ was 19.7 at baseline and 14.6 at the end of the study, with no significant difference in mean peak (*p* = 0.404). In the treatment group, the hydrogen and methane peak production showed a notable decline (*p* = 0.004) from baseline in 2 months. The mean rise in hydrogen and methane (ppm) observed was 20.8 at baseline and decreased to 5.7 after the intervention of OLT.

Table 6 reveals additional insights about symptomatic score variation within each group, showing a significant decrease in the mean score of the intervention group (*p* = 0.000). In the control group, the average symptom score of both intervals (before and after) was comparable, indicating non-significant divergence (*p* = 0.676).

**Table 6:** Within-group comparison of mean symptomatic score (SC).

Groups	Interval	Mean symptomatic score (SC)
Control	Before	4 ± 1.3
	After	3.9 ± 1.3
	*p* value	0.676
Intervention	Before	4.1 ± 1.3
	After	1.7 ± 0.9
	*p* value	0.000*

Values are expressed as mean ± S.D., Paired sample *t*-test, *level of significance (95%).

**Figure 3:**
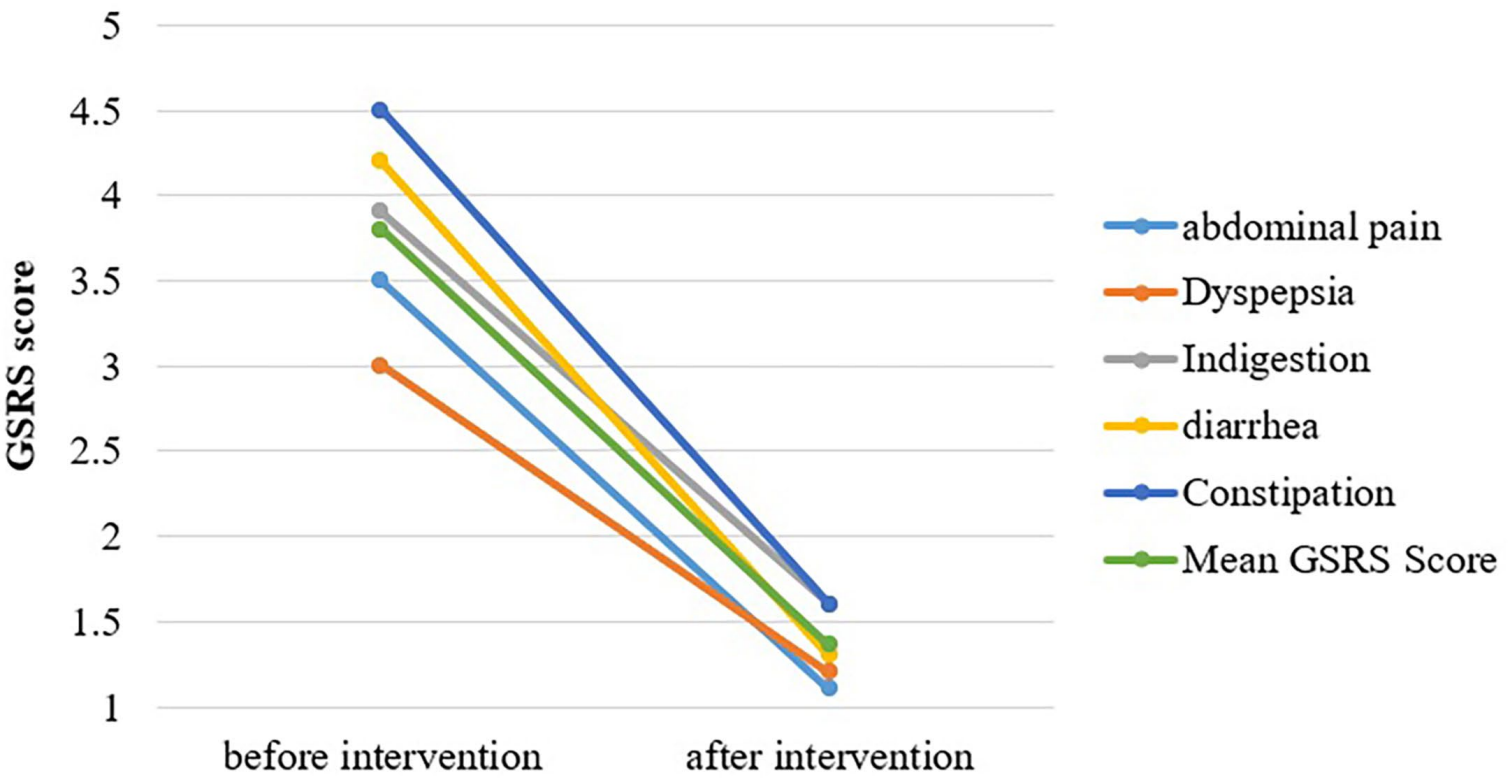
Cpmparision of GSRS score before and after intervention.

Figure 3 shows the mean of significantly heightened GI symptoms in SIBO patients. Scores for each of the five sub-dimensions of GSRS were significantly reduced after OLT intervention. The intensity of abdominal pain, hunger pains, and nausea was markedly reduced, with the mean difference being 2.4 ± 2 (*p* = 0.000). The severity of dyspepsia syndrome, including heartburn and acid reflux, reduced from a mean symptomatic score of 3.0 at baseline to 1.2 after intervention (*p* < 0.05). A considerable improvement in the abdominal distension and bloating of SIBO patients was observed with the highest mean difference of 3 (*p* = 0.000). The values of indigestion syndrome recorded at the termination point of intervention were quite lower than the baseline values, with a mean score of 4.2 and 1.3, respectively. The mean score of diarrhea syndrome falls from 4.2 (baseline) to 1.3 (post-study), with a level of significance <0.05. The intensity and frequency of constipation were largely reduced with a mean difference of 2.9 (*p* = 0.000).

## 4 Discussion

This randomized controlled trial has been conducted to observe the effectiveness of OLT intervention in normalizing hydrogen and methane concentration in GBT and in reducing GI symptoms related to SIBO, such as bloating, constipation, and abdominal pain. The effect of the intervention on the composition of different body compartments in participants was also studied.

### 4.1 Association between SIBO types and body composition variables

The results of the current study showed that most participants (53%) had a normal BMI. The link between abdominal obesity and positive GBT is also reported by Kim, Park, Paik, Kang, Jo, and Lee (15). According to another study finding targeting obese and overweight individuals, 20% of the obese patients had a positive breath test for methane (16). The pathophysiological mechanism behind SIBO and obesity can be explained by alterations in the gut microbiota composition, which may disrupt the production of appetite hormones, energy balance, and insulin secretion, potentially leading to the development of obesity (17). Our study did not reveal any significant difference in the BMI of different types of SIBO patients. Contrarily, other studies showed that the H_2_^+^/CH_4_^+^group exhibited high BMI according to Mathur, Amichai, Chua, Mirocha, Barlow, and Pimentel (18), and methane-positive patients had higher BMI (45.2 kg/m^2^) compared to methane-negative individuals (38.5 kg/m^2^) (16).

It was observed that CH^4^ producers had notably lower TBW and total protein levels compared to other groups. However, contrasting results were found in another study where the CH_4_^+^ group had higher water content than the H_2_^+^ and H_2_^+^/CH_4_^+^ groups, although this difference was not statistically significant (19). Similarly, in a separate study, the CH_4_^+^ group showed the highest SMM and body protein compared to the H_2_^+^ and H_2_^+^/CH_4_^+^ groups, but this discrepancy also lacked statistical significance. This may be explained by the fact that skeletal muscle contains a significant amount of water, and changes in hydration levels directly impact SMM and body protein. In our study, the decrease in TBW in the CH^4+^ group coincided with reduced body protein and SMM.

The results of a similar study showed that methane producers had a higher %BF than hydrogen producers (18), which comply with our study, in which the CH_4_^+^ group was observed to have the highest BFM and % body fat, but this variation was not significant. Interestingly, a study led by Wielgosz-Grochowska, Domanski, and Drywień (19) produced contrasting results to the aforementioned studies, suggesting no significant difference in the percentage of body fat between SIBO subgroups, but a trend toward a higher percentage BF was observed in the H_2_^+^ group in the present study.

Our study depicts that OLT results lead to a decrease in BMR. This observation may be linked to the fact that a substantial proportion of SIBO patients in both groups were H_2_ dominant (37%) or H_2_/CH_4_ dominant (29%), having a high BMR, leading to weight loss. Consequently, after intervention of OLT, the SIBO participants experienced a significant change in BMR, which may potentially contribute to more effective weight management and energy metabolism.

InBody analysis report of SIBO patients in a study conducted by Wielgosz-Grochowska, Domanski, and Drywień (19) showed similarities in bone mineral mass across all SIBO types, with the methane-dominant group showing the highest mineral content compared to other groups. Contrarily, our study revealed the highest bone mass in the H_2_^+^ group, followed by the H_2_/CH_4_ group, with the methane group showing the least concentration of bone minerals. Another study finding revealed that SIBO is associated with low bone mineral density, suggesting SIBO might be the reason behind unexplained osteopenia (20).

### 4.2 The GI symptoms assessed by the mean SC and GSRS questionnaire

Many functional foods and plants with therapeutic properties manage gut-related disorders and symptoms. The results of this study support the hypothesis that herbal therapy addressed the GI symptoms better than antibiotic therapy. The frequency and intensity of the GI symptoms, GI discomfort (*p* < 0.001), and overall GSAS (GERD Symptom Assessment Scale) score are considerably improved by taking *O. europaea* leaves extract (21). Comparative analysis with our study showed that the GI symptoms of SIBO patients were reduced significantly after OLT intervention. While 7-day rifaximin (antibiotic) therapy (22) only decreases the intensity of diarrhea, rumbling sounds in the stomach, and lethargy, no improvement is reported in the severity of anorexia, abdominal pain, and nausea.

Better outcomes were observed in SIBO patients taking broad-spectrum antibiotic therapy combined with probiotic therapy (Lactol) in the rehabilitation phase, with significant alleviation in symptoms of abdominal pain, flatulence, burping, loose stools, and fecal urgency (23). In contrast, OLT turned out to be more effective than above mentioned antibiotics in decreasing the severity of documented symptoms.

In our study, the intensity, frequency, and duration of constipation syndrome have significantly reduced after OLT intervention (*p* = 0.000) compared to the control group. Comparatively, a trial has been conducted to check the effect of an herbal mix (ginger, ginseng, and Japanese pepper) to treat functional constipation. Both ginger and olive leaf contain polyphenols, flavonoids, and terpenes, which exhibit antioxidant and antibacterial properties. Moreover, olive leaf and ginseng also share some bioactive components, including polysaccharides, saponins, sterols, and polyphenols. The herbal mix was found to increase peristaltic contractions and colon transit activity (24).

The OLT consumption for 2 months resulted in 87.5% GBT normalization rates, which is comparatively higher than the elemental diet, 80–85% (25). The antibiotic therapy combined with probiotic therapy (Lactol) has a higher GBT normalization rate (93.3%) than taking antibiotics (66.7%) (23) and probiotics alone (55%) (26).

## 5 Conclusion

The findings of this investigation have unveiled the therapeutic benefits of OLT against SIBO by alleviating the intrusiveness, frequency, and duration of GI symptoms. The efficacy of OLT is underscored by its ability to normalize GBT results by decreasing H_2_ and CH_4_ concentrations during the breath test. Nevertheless, the body composition parameters did not show much improvement throughout the study period. The intervention group experienced a decrease in TBW, which ultimately affects BMR, % muscle mass, and % fat mass, although BFM and BMD did not change. It is noteworthy that H_2_^+^/CH_4_^+^ SIBO patients have higher levels of protein, bone minerals, muscle mass, and BMR compared to H_2_^+^ and CH_4_^+^ groups alone. Beyond its therapeutic aspect, this study also presents evidence that SIBO is associated with an increased risk of obesity.

## Data Availability

The raw data supporting the conclusions of this article will be made available by the authors, without undue reservation.

## References

[1] Rezaie A Pimentel M Rao SS. How to test and treat small intestinal bacterial overgrowth: an evidence-based approach. Curr Gastroenterol Rep. (2016) 18:8. doi: 10.1007/s11894-015-0482-9 26780631

[2] Erdogan A Rao SSC Gulley D Jacobs C Lee Y Badger C. Small intestinal bacterial overgrowth: duodenal aspiration vs glucose breath test. Neurogastroenterol Motil. (2015) 27:481–9. doi: 10.1111/nmo.12516, PMID: 25600077

[3] Elphick DA Chew TS Higham SE Bird N Ahmad A Sanders DS. Small bowel bacterial overgrowth in symptomatic older people: can it be diagnosed earlier? Gerontology. (2005) 51:396–401. doi: 10.1159/000088704, PMID: 16299421

[4] Skrzydło-Radomańska B Cukrowska BJJ o CM. How to recognize and treat small intestinal bacterial overgrowth? J Clin Med. (2022) 11:6017. doi: 10.3390/jcm11206017, PMID: 36294338 PMC9604644

[5] Sheeza I Iqbal S Rabbani I Ali MA. Prevalence of small intestinal bacterial overgrowth in people with gastrointestinal signs and symptoms using glucose breath test. Act Sci Nutr Health. (2021) 5:127–37.

[6] Onana Ndong P Boutallaka H Marine-Barjoan E Ouizeman D Mroue R Anty R. Prevalence of small intestinal bacterial overgrowth in irritable bowel syndrome (IBS): correlating H2 or CH4 production with severity of IBS. JGH Open. (2023) 7:311–20. doi: 10.1002/jgh3.12899, PMID: 37125253 PMC10134763

[7] Gandhi A Shah A Jones MP Koloski N Talley NJ Morrison M. Methane-positive small intestinal bacterial overgrowth in inflammatory bowel disease and irritable bowel syndrome: a systematic review and meta-analysis. Gut Microbes. (2021) 13:1933313. doi: 10.1080/19490976.2021.1933313, PMID: 34190027 PMC8253120

[8] Sachdev AH Pimentel M. Gastrointestinal bacterial overgrowth: pathogenesis and clinical significance. Ther Adv Chronic Dis. (2013) 4:223–31. doi: 10.1177/2040622313496126, PMID: 23997926 PMC3752184

[9] Ren X Di Z Zhang Z Fu B Wang Y Huang C. Chinese herbal medicine for the treatment of small intestinal bacterial overgrowth (SIBO): a protocol for systematic review and meta-analysis. Medicine. (2020) 99:e23737. doi: 10.1097/md.0000000000023737, PMID: 33371127 PMC7748159

[10] Baldoni L Belaj A. Olive In: Vollmann J Rajcan I, editors. Oil crops. New York, NY: Springer (2010). 397–421.

[11] Rocchetti G Callegari LM Senizza A Giuberti G Ruzzolini J Romani A. Oleuropein from olive leaf extracts and extra-virgin olive oil provides distinctive phenolic profiles and modulation of microbiota in the large intestine. Food Chem. (2022) 380:132187. doi: 10.1016/j.foodchem.2022.132187 35086016

[12] Farràs M Martinez-Gili L Portune K Arranz S Frost G Tondo M. Modulation of the gut microbiota by olive oil phenolic compounds: implications for lipid metabolism, immune system, and obesity. Nutrients. (2020) 12:2200. doi: 10.3390/nu12082200, PMID: 32718098 PMC7468985

[13] Cör Andrejč D Butinar B Knez Ž Tomažič K Knez Marevci M. The effect of drying methods and extraction techniques on Oleuropein content in olive leaves. Plants. (2022) 11:865. doi: 10.3390/plants11070865, PMID: 35406845 PMC9003305

[14] Ramírez EM Brenes M Romero C Medina E. Olive leaf processing for infusion purposes. Foods. (2023) 12:591. doi: 10.3390/foods12030591, PMID: 36766119 PMC9914354

[15] Kim DB Park CS Paik CN Kang YJ Jo IH Lee JM. Relationship between untreated obstructive sleep apnea and breath hydrogen and methane after glucose load. Saudi J Gastroenterol. (2022) 28:355–61. doi: 10.4103/sjg.sjg_134_22, PMID: 35848702 PMC9752531

[16] Basseri RJ Basseri B Pimentel M Chong K Youdim A Low K. Intestinal methane production in obese individuals is associated with a higher body mass index. Gastroenterol Hepatol. (2012) 8:22–8. PMC3277195 22347829

[17] Yao Q Yu Z Meng Q Chen J Liu Y Song W. The role of small intestinal bacterial overgrowth in obesity and its related diseases. Biochem Pharmacol. (2023) 212:115546. doi: 10.1016/j.bcp.2023.115546, PMID: 37044299

[18] Mathur R Amichai M Chua KS Mirocha J Barlow GM Pimentel M. Methane and hydrogen positivity on breath test is associated with greater body mass index and body fat. J Clin Endocrinol Metab. (2013) 98:E698–702. doi: 10.1210/jc.2012-3144, PMID: 23533244 PMC3615195

[19] Wielgosz-Grochowska JP Domanski N Drywień ME. Influence of body composition and specific anthropometric parameters on SIBO type. Nutrients. (2023) 15:4035. doi: 10.3390/nu15184035, PMID: 37764818 PMC10535553

[20] Stotzer PO Johansson C Mellström D Lindstedt G Kilander AF. Bone mineral density in patients with small intestinal bacterial overgrowth. Hepato-Gastroenterology. (2003) 50:1415–8. 14571751

[21] Malfa GA Di Giacomo C Cardia L Sorbara EE Mannucci C Calapai G. A standardized extract of *Opuntia ficus-indica* (L.) mill and *Olea europaea* L. improves gastrointestinal discomfort: a double-blinded randomized-controlled study. Phytother Res. (2021) 35:3756–68. doi: 10.1002/ptr.7074, PMID: 33724592

[22] Di Stefano M Malservisi S Veneto G Ferrieri A Corazza GR. Rifaximin versus chlortetracycline in the short-term treatment of small intestinal bacterial overgrowth. Aliment Pharmacol Ther. (2000) 14:551–6. doi: 10.1046/j.1365-2036.2000.00751.x 10792117

[23] Khalighi AR Khalighi MR Behdani R Jamali J Khosravi A Kouhestani S. Evaluating the efficacy of probiotic on treatment in patients with small intestinal bacterial overgrowth (SIBO)--a pilot study. Indian J Med Res. (2014) 140:604–8. 25579140 PMC4311312

[24] Kubota K Mase A Matsushima H Fujitsuka N Yamamoto M Morine Y. Daikenchuto, a traditional Japanese herbal medicine, promotes colonic transit by inducing a propulsive movement pattern. Neurogastroenterol Motil. (2019) 31:e13689. doi: 10.1111/nmo.13689, PMID: 31374154 PMC6852043

[25] Pimentel M Constantino T Kong Y Bajwa M Rezaei A Park S. A 14-day elemental diet is highly effective in normalizing the lactulose breath test. Dig Dis Sci. (2004) 49:73–7. doi: 10.1023/b:ddas.0000011605.43979.e1, PMID: 14992438

[26] García-Collinot G Madrigal-Santillán EO Martínez-Bencomo MA Carranza-Muleiro RA Jara LJ Vera-Lastra O. Effectiveness of *Saccharomyces boulardii* and metronidazole for small intestinal bacterial overgrowth in systemic sclerosis. Dig Dis Sci. (2020) 65:1134–43. doi: 10.1007/s10620-019-05830-0, PMID: 31549334

